# The combined effect of cardiorespiratory and muscular fitness on the incidence of metabolic syndrome before midlife

**DOI:** 10.1002/jcsm.13503

**Published:** 2024-06-07

**Authors:** Kun‐Zhe Tsai, Chen‐Chih Chu, Wei‐Chun Huang, Xuemei Sui, Carl J. Lavie, Gen‐Min Lin

**Affiliations:** ^1^ Department of Medicine Hualien Armed Forces General Hospital Hualien Taiwan; ^2^ Department of Stomatology of Periodontology Mackay Memorial Hospital Taipei Taiwan; ^3^ Department of Periodontology School of Dentistry, National Defense Medical Center and Tri‐Service General Hospital Taipei Taiwan; ^4^ Department of Medicine Tri‐Service General Hospital and National Defense Medical Center Taipei Taiwan; ^5^ College of Medicine National Yang Ming Chiao Tung University Taipei Taiwan; ^6^ Department of Critical Care Medicine Kaohsiung Veterans General Hospital Kaohsiung Taiwan; ^7^ Department of Exercise Science, Arnold School of Public Health University of South Carolina Columbia SC USA; ^8^ John Ochsner Heart and Vascular Institute, Ochsner Clinical School University of Queensland School of Medicine New Orleans LA USA

**Keywords:** Cardiorespiratory fitness, Cohort study, Metabolic syndrome, Muscular endurance capacity, Young adults

## Abstract

**Background:**

Cardiorespiratory fitness (CRF) could reduce the risk of metabolic syndrome (MetS) while the association between muscular endurance capacity (MEC) and incident MetS has rarely been investigated in young adults.

**Methods:**

A total of 2890 military men and women, aged 18–39 years, free of baseline MetS in Taiwan, were followed for incident MetS from baseline (2014) until the end of 2020. All subjects received annual health examinations for assessment of MetS. Physical fitness was assessed by CRF (estimated maximal oxygen uptake, VO_2_ max [mL/kg/min], in a 3000‐m run) and MEC (numbers of 2‐min push‐ups). MetS was defined according to the International Diabetes Federation (IDF) criteria. Multiple Cox regression analysis was conducted with adjustments for baseline age, sex, substance use status and physical activity to determine the associations of CRF and MEC with incidences of new‐onset MetS and related features, for example, central obesity, hypertension, dyslipidaemia and prediabetes or diabetes. To examine the combined effects of CRF and MEC status on incidence of MetS, high and low levels of CRF and MEC were separately defined by over and under the sex‐specific median in each exercise test.

**Results:**

During a median follow‐up of 5.8 years, there were 673 (23.3%) new‐onset MetS. Higher CRF was associated with a lower incidence of MetS (hazard ratio [HR] and 95% confidence interval: 0.905 [0.877–0.933]), and its components separately, except hypertension. No association was observed between MEC and incident MetS, and its components separately, except hypertension. When evaluating the combined effects of MEC and CRF status on the incidence of MetS, it was observed that compared with the low CRF/low MEC, the high CRF/high MEC (HR: 0.553 [0.439–0.697]) and the high CRF/low MEC (HR: 0.730 [0.580–0.918]) had a lower incidence of new‐onset MetS (*P* value for the intergroup difference = 0.04). There was no significant result for the low CRF/high MEC.

**Conclusions:**

This study highlights that although the protective effects of MEC to reduce the incidence of MetS and most of its related features were mainly driven by CRF in young adults, there was an addictive effect of greater MEC on CRF to prevent the development of new‐onset MetS before midlife.

## Introduction

Metabolic syndrome (MetS) is characterized by a cluster of metabolic disorders, that is, central obesity, hypertension, dyslipidaemia and elevated blood glucose, which significantly increase the risk of cardiovascular disease (CVD) and all‐cause death.^1^, ^2^ The global prevalence of MetS ranges 3.3–19.2%, with estimates of 12–37% in the Asian populations.^3^ Among 20 leading global risk factors for years of life lost in 2040, metabolic disorders are anticipated to rank as the foremost risk variables.^1^ This has led to increased scrutiny and interest within the scientific community for the notion of metabolic health. A mounting body of research, including recent clinical trials, consistently demonstrated that greater physical activity (PA) such as aerobic and resistance exercise training is inversely associated with the risk of MetS.^2^, ^4^, ^5^ With regard to physical fitness, cardiorespiratory fitness (CRF) and muscular strength have revealed beneficial effects on reducing metabolic abnormalities and incident CVD events in those with MetS or diabetes, including in young adults.^6^, ^7^, ^8^ However, prior evidence for CRF and muscular fitness with the risk of metabolic disorders had rarely taken PA levels into account, and most were from cross‐sectional studies.^8^, ^9^, ^10^ As PA level is a crucial contributor to MetS, the effect of physical fitness on the risk of MetS should be clarified with an adjustment for the baseline PA levels.^10^

Most studies on MetS have emphasized on treatment strategies, whereas there was a noticeable gap in research for primary prevention of MetS. To the best of our knowledge, an association between combined CRF and muscular strength and prevalent MetS has been found in prior cross‐sectional studies,^11^, ^12^, ^13^ while no cohort studies were available to affirm the temporal association. Because both MEC and CRF can increase insulin sensitivity and glucose tolerance and reduce abdominal fat and weight,^14^, ^15^ we hypothesized that a combination of MEC and CRF levels may predict the development of MetS. Therefore, this cohort study was aimed to examine the associations of CRF and MEC with the incidences of new‐onset MetS and its related features among young adults.

## Methods

### Study population

The cohort study included 4080 military personnel, aged 18–50 years, from the cardiorespiratory fitness and health in eastern armed forces (CHIEF) study in Taiwan at the beginning in 2014.^16^, ^17^, ^18^ Each participant underwent annual health examinations for evaluating the new‐onset MetS events. The study design and protocol has been reviewed and approved by the Institutional Review Board (IRB) of the Mennonite Christian Hospital (certificate No. 16‐05‐008) in Hualien, Taiwan. Written informed consent was obtained from all participants.

### Annual health examinations (2014–2020)

Anthropometric variables of waist circumference (WC), body height and body weight were measured in the standing position. Body mass index (BMI) was calculated as the body weight (kg) divided by square of the body height (m^2^). The resting blood pressure (BP) of each participant was measured once over the right arm in the sitting position, using the oscillometric method through the automatic BP device (FT201 Parama‐Tech Co., Ltd, Fukuoka, Japan).^19^, ^20^ If the initial BP level was ≥130/80 mmHg, the BP had to be measured for a second time after a rest for 15 min. The final BP level was an average of the initial and the second BP levels. Serum triglycerides (TG), total cholesterol, high‐density lipoprotein cholesterol (HDL‐C) and fasting plasma glucose (FPG) were measured from an overnight 12‐h fasting blood sample of each subject by the auto analyser (Olympus AU640, Kobe, Japan).

Substance use status, that is, alcohol consumption, betel nut chewing and tobacco smoking were self‐reported by participants as active or former/never. Betel nut chewing is prevalent in Southeastern Asian populations and has been observed to increase the risk of MetS and reduce CRF and MEC.^21^ Moderate‐intensity PA assessed by leisure‐time running (<150, 150–299 and ≥300 min/week) in the past half a year were self‐administered in a questionnaire in the Hualien Armed Forces General Hospital at baseline (2014).^22^ All participants underwent the exercise tests for assessments of CRF by maximal oxygen uptake (VO_2_ max, mL/kg/min) in a 3000‐m run and MEC by 2‐min push‐up numbers at the Hualien Military Physical Training and Testing Center at baseline.^23^, ^24^

### Baseline physical fitness assessments (2014)

Both MEC and CRF of each subject were assessed in the afternoon (2:30 pm–4:30 pm) in the same day. MEC of each subject was evaluated by 2‐min push‐up numbers performed in the main building of the Hualien Military Physical Training and Testing Center where the indoor humidity and temperature were controlled constantly by the air conditioners. Push‐up numbers in 2 min were scored by infrared sensors, and the test was prematurely stopped if the trunk touched down the ground. CRF of each subject was evaluated in a 3000‐m run field test, which was uniformly performed outdoor at 4:00 pm if the weather situation was acceptable according to the military testing regulation.^18^, ^23^ Time for a 3000‐m run was transformed to estimated VO_2_ max, which has been verified by the cardiopulmonary exercising test, according to our previous work.^24^, ^25^ The entire procedure for both of the 2‐min push‐up and 3000‐m run field tests was video recorded throughout.

### Definition of incident MetS events (2015–2020)

MetS was defined based on the International Diabetes Federation (IDF) criteria specifically for Chinese,^26^ as having three or more of the following clinical features: (1) serum TG ≥ 150 mg/dL, or with lipid‐lowering medications; (2) HDL‐C < 50 mg/dL for women and <40 mg/dL for men; (3) WC ≥ 80 cm for women and ≥90 cm for men; (4) FPG ≥ 100 mg/dL, or with anti‐diabetic medications; (5) systolic BP ≥ 130 mmHg, or diastolic BP ≥ 85 mmHg, or with antihypertensive medications.

### Statistical analysis

Baseline characteristics of the young military cohort were presented as mean ± standard deviation (*SD*) and numbers (percentage) for continuous variables and categorical variables, respectively. Follow‐up of each subject was started at baseline (2014) and continued until the first occurrence of MetS events, loss to follow‐up, and at the end of follow‐up (31 December 2020).

To determine the survival probability, we utilized Kaplan–Meier curve analysis for the sex‐specific tertiles. The 1st CRF tertile was VO_2_ max ≤33.44 mL/min/kg in men and ≤27.90 mL/min/kg in women; the 2nd CRF tertile was 33.45–35.15 mL/min/kg in men and 27.91–29.50 mL/min/kg in women; and the 3rd CRF tertile was >35.15 mL/min/kg in men and >29.50 mL/min/kg in women. The MEC 1st tertile was 2‐min push‐up numbers ≤48 in men and ≤33 in women; the 2nd MEC tertile was 49–51 in men and 34–37 in women; and the 3rd MEC tertile was >51 in men and >37 in women. Differences between the sex‐specific tertiles were compared by log‐rank test.

To determine the association between physical fitness and incidence of MetS, we used multivariable Cox regression analysis with simultaneous adjustments for baseline age, sex, alcohol intake status, betel nut chewing status, smoking status and PA (Model 1). MEC and CRF were treated separately as continuous variables (each increase of 1 unit), as well as categorical variables by sex‐specific tertiles. Model 2 consisted of an additional adjustment for MEC in the analysis with CRF and for CRF in the analysis with MEC.

To explore the combined effects of CRF and MEC on the incidence of MetS, we used multivariable Cox regression analysis with simultaneous adjustments for the covariates in Model 1. Participants were divided into four groups according to CRF and MEC status: ‘High CRF/High MEC ‘, ‘High CRF/Low MEC’, ‘Low CRF/High MEC’ and ‘Low CRF/Low MEC’. High and low levels of CRF and MEC were separately categorized by over and under the sex‐specific median in each exercise test. The median number of the 2‐min push‐up test was 50 in men and 35 in women, and the median level of VO_2_ max in the 3000‐m run test was 34.2 mL/min/kg in men and 25.6 mL/min/kg in women.

To determine the associations of MEC and CRF (treated as continuous variables) with the incidence of five MetS features including central obesity, hypertension, dyslipidaemia and prediabetes/diabetes, we used multivariable Cox regression analysis with simultaneous adjustments for the covariates in Model 2.

The covariates were selected in the multivariable Models mainly according to their associations with physical fitness and MetS in prior studies.^17^, ^21^ A value of *P* < 0.05 was regarded as statistically significant. All statistical analyses were performed using the software of SPSS v25.0 for Windows (IBM Corp., Armonk, NY, USA).

## Results

### Baseline characteristics of the study population

Participants were excluded for the presence of baseline MetS (*N* = 457), age ≥40 years (*N* = 58) and those who moved out of the military bases in Eastern Taiwan and thus lost follow‐up for the new‐onset MetS, leaving a final sample of 2890 subjects for analysis. The baseline characteristics of the study cohort are shown in *Table* *1*. The participants were aged 28.38 ± 5.79 years on average at baseline. Of them, 2581 (89.3%) were men and 309 (10.7%) were women. There were 80% of participants with moderate‐intensity PA levels ≥150 min/week. Over a median follow‐up period of 5.8 years, 673 participants (23.3%) developed MetS.

**Table 1:** Baseline characteristics of the study cohort

	*N* = 2890
Age, years	28.38 ± 5.79
Sex, %	
Men	2581 (89.3)
Women	309 (10.7)
Lifestyle behaviour, %	
Alcohol drinking	1161 (40.2)
Betel nut chewing	279 (9.8)
Tobacco smoking	991 (34.7)
Body mass index, kg/m^2^	24.27 ± 3.00
Waist circumference, cm	81.44 ± 8.06
Systolic BP, mmHg	115.89 ± 12.90
Diastolic BP, mmHg	69.30 ± 9.64
Blood test	
Total cholesterol, mg/dL	171.98 ± 32.56
LDL‐C, mg/dL	103.83 ± 28.96
HDL‐C, mg/dL	49.93 ± 10.01
Serum triglycerides, mg/dL	96.93 ± 58.73
Fasting glucose, mg/dL	91.99 ± 9.73
PA level, %	
<150 min/week	627 (21.7)
150–299 min/week	1104 (38.2)
≥300 min/week	1159 (40.1)
2‐min push‐up numbers	48.16 ± 11.94
VO_2_ max, mL/min/kg	33.63 ± 3.12

*Note*: Data are presented as numbers (%) and mean (standard deviation).

Abbreviations: BP, blood pressure; HDL‐C, high‐density lipoprotein cholesterol; LDL‐C, low‐density lipoprotein cholesterol; PA, physical activity; VO_2_ max, maximal oxygen uptake.

### Physical fitness and incident MetS in the overall cohort

**Figure 1:**
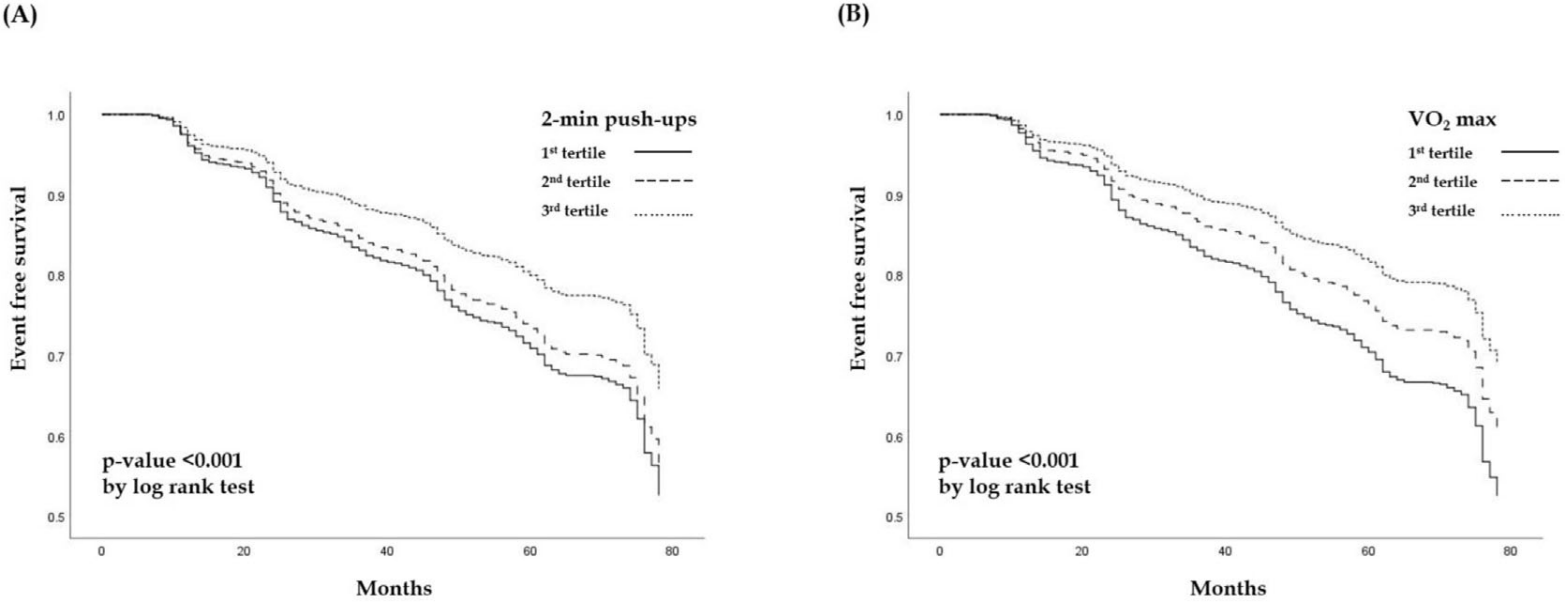
The Kaplan–Meier curve survival analysis results demonstrate that those with greater muscular endurance capacity (*A*) and cardiorespiratory fitness (*B*) have a lower incidence of new‐onset metabolic syndrome while compared with their counterparts (both *P* < 0.001).

*Figure* *1* reveals the Kaplan–Meier curve analysis results, in which those with greater MEC and CRF have a lower incidence of MetS events while compared with those with lower MEC and CRF (both *P* < 0.01 by log rank test). *Table* *2* shows the results of multivariable Cox regression analysis investigating the new‐onset MetS events while considering physical fitness. Both 2‐min push‐up numbers and VO_2_ max, which were treated as continuous variables, revealed an inverse association with the incidence of new‐onset MetS (hazard ratios [HRs] and 95% confidence intervals: 0.984 [0.977–0.991] and 0.905 [0.877–0.933], respectively) in Model 1. If 2‐min push‐ups and VO_2_ max were separately treated as categorical variables by their sex‐specific tertiles, the highest tertiles were associated with a lower risk of new‐onset MetS (HRs: 0.650 [0.524–0.806] and 0.579 [0.459–0.729], respectively) as compared with their lowest tertiles. While both MEC and CRF were adjusted simultaneously in Model 2, the association of MEC (continuous variable) with incident MetS became not significant (HR: 0.997 [0.988–1.007]). On the contrary, the association of CRF (continuous variable) with incidence of MetS remained significant (HR: 0.907 [0.876–0.939]). When MEC and CRF were treated as categorical variables by sex‐specific tertiles, the findings were in line with those treated as continuous variables.

**Table 2:** Multivariable Cox regression analysis for incidence of new‐onset metabolic syndrome before midlife with physical fitness in military adults

			Unadjusted model			Model 1^a^			Model 2^b^		
Physical performances	*N*	Events	HR	95% CI	*P* value	HR	95% CI	*P* value	HR	95% CI	*P* value
2‐min push‐ups^c^											
Continuous variable	2890	673	0.992	0.986–0.998	0.01	0.984	0.977–0.991	<0.001	0.997	0.988–1.007	0.58
1st tertile	899	265	1.000			1.000			1.000		
2nd tertile	922	235	0.739	0.619–0.884	0.001	0.902	0.749–1.086	0.27	1.112	0.906–1.366	0.31
3rd tertile	1069	173	0.478	0.393–0.580	<0.001	0.650	0.524–0.806	<0.001	0.880	0.691–1.120	0.29
*P* value for trend					<0.001			<0.001			0.31
VO_2_ max, mL/min/kg^d^											
Continuous variable	2890	673	0.961	0.938–0.985	0.001	0.905	0.877–0.933	<0.001	0.907	0.876–0.939	<0.001
1st tertile	957	296	1.000			1.000			1.000		
2nd tertile	949	227	0.694	0.576–0.838	<0.001	0.771	0.635–0.937	0.009	0.779	0.639–0.949	0.01
3rd tertile	984	150	0.457	0.370–0.564	<0.001	0.579	0.459–0.729	<0.001	0.606	0.476–0.771	<0.001
*P* value for trend					<0.001			<0.001			<0.001

*Note*: Data are presented as hazard ratio (HR) and 95% confidence interval (CI).

^a^ Multiple Cox regression Model 1 adjusted for age, sex, alcohol drinking status, betel nut chewing status, tobacco smoking status and moderate‐intensity physical activity levels.

^b^ Multiple Cox regression Model 2 adjusted for Model 1 covariates + VO_2_ max + 2‐min push‐up numbers.

^c^ For 2‐min push‐up numbers, the 1st tertile was ≤48 in men and ≤33 in women; the 2nd tertile was 49–51 in men and 34–37 in women; the 3rd tertile was >51 in men and >37 in women.

^d^ For VO_2_ max, the 1st tertile was ≤33.44 mL/min/kg in men and ≤27.90 mL/min/kg in women; the 2nd tertile was 33.45–35.15 mL/min/kg in men and 27.91–29.50 mL/min/kg in women; the 3rd tertile was >35.15 mL/min/kg in men and >29.50 mL/min/kg in women.

### Combined effects of MEC and CRF status on incidence of MetS

*Table* *3* shows the results of the associations of combined MEC/CRF status with new‐onset MetS. As compared with the low CRF/low MEC group, there were graded lower incidences of new‐onset MetS in the low CRF/high MEC, high CRF/low MEC and high CRF/Hhigh MEC groups in the crude model (HRs: 0.696 [0.553–0.875], 0.654 (0.523–0.819) and 0.387 (0.317–0.473), respectively; *P* value for trend <0.001). In the multivariable model, the high CRF/high MEC group remained with the lowest incidence of new‐onset MetS (HR: 0.553 [0.439–0.697]), which was found significantly lower than the high CRF/low MEC and low CRF/high MEC groups (HRs: 0.730 [0.580–0.918] and 0.841 [0.662–1.069], respectively; *P* value for intergroup difference = 0.04 and 0.002, respectively).

**Table 3:** Associations of cardiorespiratory fitness and muscular endurance capacity status with incident metabolic syndrome before midlife

			Unadjusted model				Multivariable model 1^b^			
CRF/MEC status^a^	*N*	Events	HR	95% CI	*P* value	*P* value for interaction	HR	95% CI	*P* value	*P* value for interaction
Low MEC/low CRF	1082	345	1.000				1.000			
High MEC/low CRF	390	93	0.696	0.553–0.875	0.002	0.67^c^	0.841	0.662–1.069	0.15	0.33^c^
Low MEC/high CRF	429	99	0.654	0.523–0.819	<0.001	<0.001^d^	0.730	0.580–0.918	0.007	0.04^d^
High MEC/high CRF	989	136	0.387	0.317–0.473	<0.001	<0.001^e^	0.553	0.439–0.697	<0.001	0.002^e^
*P* value for trend					<0.001				<0.001	

*Note*: Data are presented as hazard ratio (HR) and 95% confidence interval (CI).

Abbreviations: CRF: cardiorespiratory fitness; MEC, muscular endurance capacity.

^a^ ‘High CRF’ was defined as VO_2_ max > 34.2 mL/min/kg in men and >25.6 mL/min/kg in women which was the greater half of CRF. ‘Low CRF’ was defined as VO_2_ max ≤ 34.2 mL/min/kg in men and ≤25.6 mL/min/kg in women which was the lower half of CRF. ‘High MEC’ was defined as 2‐min push‐up numbers >50 in men and >35 in women which was the greater half of MEC. ‘Low MEC’ was defined as 2‐min push‐up numbers ≤50 in men and ≤35 in women which was the lower half of MEC.

^b^ Multivariable Model 1: age, sex, alcohol drinking status, betel nut chewing status, tobacco smoking status and moderate‐intensity physical activity levels adjustments.

^c^ High MEC/low CRF versus low MEC/high CRF.

^d^ Low MEC/high CRF versus high MEC/high CRF.

^e^ High MEC/low CRF versus high MEC/high CRF.

### Associations of MEC and CRF with incidence of each MetS feature

*Table* *4* reveals the results of multivariable Cox regression analysis for the risk of each new‐onset MetS feature with MEC and CRF, respectively. Greater MEC and CRF were associated with a lower incidence of central obesity (HRs: 0.982 [0.975–0.990] and 0.893 [0.864–0.923], respectively), hypertension (HRs: 0.984 [0.976–0.993] and 0.949 [0.913–0.986], respectively), hypertriglyceridaemia (HRs: 0.985 [0.978–0.993] and 0.910 [0.879–0.942], respectively) and low HDL‐C (HRs: 0.986 [0.977–0.995] and 0.930 [0.893–0.970], respectively) in Model 1. However, for diabetes or prediabetes, a lower risk of new‐onset MetS was only observed with higher CRF but not with higher MEC (HRs: 0.942 [0.906–0.979] and 0.995 [0.986–1.004], respectively). When MEC and CRF were simultaneously adjusted in Model 2, the association between CRF and the incidence of each MetS feature remained significant except new‐onset hypertension (HR: 0.968 [0.927–1.010]). However, most of the associations of MEC with MetS related features were not significant except new‐onset hypertension (HR: 0.988 [0.978–0.999]).

**Table 4:** Associations of cardiorespiratory fitness and muscular endurance capacity with incidence of each metabolic syndrome feature before midlife in military adults

			2‐min push‐up numbers						VO_2_ max					
			Model 1^a^			Model 2^b^			Model 1^a^			Model 2^b^		
MetS features	N*^c^	Events	HR	95% CI	*P* value	HR	95% CI	*P* value	HR	95% CI	*P* value	HR	95% CI	*P* value
Central obesity	2388	542	0.982	0.975–0.990	<0.001	0.993	0.983–1.002	0.13	0.893	0.864–0.923	<0.001	0.903	0.870–0.937	<0.001
Hypertension	2434	476	0.984	0.976–0.993	<0.001	0.988	0.978–0.999	0.02	0.949	0.913–0.986	0.007	0.968	0.927–1.010	0.13
Hypertriglyceridaemia	2553	551	0.985	0.978–0.993	<0.001	0.995	0.985–1.004	0.27	0.910	0.879–0.942	<0.001	0.918	0.883–0.954	<0.001
Low HDL‐C	2457	377	0.986	0.977–0.995	0.002	0.995	0.983–1.006	0.37	0.930	0.893–0.970	0.001	0.939	0.896–0.984	0.008
Prediabetes/diabetes	2530	503	0.995	0.986–1.004	0.24	1.004	0.993–1.016	0.42	0.942	0.906–0.979	0.003	0.935	0.896–0.976	0.002

*Note*: Data are presented as hazard ratio (HR) and 95% confidence interval (CI). Definitions: Hypertension was defined as systolic blood pressure ≥130 mmHg and/or diastolic blood pressure≥85 mmHg, or with antihypertensive medications therapy; central obesity was defined as waist circumference ≥90 cm in men and ≥80 cm in women; prediabetes/diabetes was defined as fasting glucose >100 mg/dL or on antidiabetic medications therapy; low HDL‐C was defined as <40 mg/dL in men and <50 mg/dL in women; hypertriglyceridaemia was defined as fasting serum triglycerides ≥150 mg/dL.

Abbreviations: HDL‐C, high‐density lipoprotein cholesterol; MetS, metabolic syndrome, VO_2_ max, maximal oxygen uptake.

^a^ Multiple Cox regression Model 1 adjusted for age, sex, alcohol drinking status, betel nut chewing status, tobacco smoking status, and moderate‐intensity physical activity levels.

^b^ Multiple Cox regression Model 2 adjusted for Model 1 covariates + VO_2_ max + 2‐min push‐up number.

^c^ N* indicates that the sample size for analysis for the new‐onset specific MetS feature excluded those with that MetS feature from the overall cohort (*N* = 2890) at baseline.

## Discussion

The principal findings of this study were that among young military adults, the greater CRF condition, independent of MEC, is what determines the lower risk of incidence of MetS, and that when combined, there is an addictive effect of greater MEC on CRF to reduce the incidence of new‐onset MetS. The beneficial effect of MEC is mainly attributed to a lower risk of incidence of hypertension, which is independent of CRF.

In many of the prior studies,^27^, ^28^, ^29^, ^30^, ^31^, ^32^ MetS was defined using the third report of the National Cholesterol Education Program (NCEP) Adult Treatment Panel (ATP III) criteria,^33^ and CRF was evaluated by maximal metabolic equivalents obtained from a treadmill test. For prior cross‐sectional studies,^27^, ^28^ an inverse association between CRF and prevalent MetS was found in middle‐aged adults. With regard to cohort studies, a study involving 10 498 men and women with a mean age of 44 years revealed that the lowest CRF tertile was an independent predictor for the incident MetS during a mean follow‐up period of 5.7 years.^29^ Another study including 3411 men with a mean age of 42.3 years revealed a consistent reduction in the risk of all MetS features with greater CRF quintiles while compared with the lowest CRF quintile for a mean follow‐up period of 9.0 years.^30^ In some randomized clinical trials,^31^, ^32^ increased CRF related to aerobic exercise training was found to reduce metabolic disorders among middle‐aged individuals with baseline MetS or diabetes. However, only a few cohort studies were available for young adults. Consistent with our study findings, Carnethon et al. revealed that greater a CRF was associated with a lower incidence of new‐onset diabetes, hypertension and MetS among 4487 young adults, aged 18–30 years, for a mean follow‐up period of 15 years in the Coronary Artery Risk Development in Young Adults (CARDIA) study.^7^

Also, there were many cross‐sectional studies revealing an inverse association of muscular strength assessed by a variety of exercise modalities with prevalent MetS among young and middle‐aged individuals.^11^, ^12^, ^13^ Aligned with these study findings, a combined effect of higher CRF and muscular strength demonstrated graded lower risk for MetS in these cross‐sectional studies.^12^, ^13^ The magnitude of the association of prevalent MetS with muscular strength was also found to be lower than that with CRF and was attenuated with simultaneously adjustments for CRF.^12^, ^13^ However, there were only a few cohort studies mainly for middle‐ and old‐aged individuals. Shen et al. revealed that in a cohort of 3350 Chinese individuals with a mean age of 60.0 years, while compared with the highest handgrip strength quartile, the lowest handgrip strength quartile was associated with a higher risk of MetS in both men and women (HRs: 1.76 [1.12–2.78] and 1.28 [1.03–1.55], respectively) during a median follow‐up period of 4.0 years.^34^ In another cohort study for 3233 men aged 20–80 years, Jurca et al. demonstrated that the highest muscular strength quartile evaluated by leg and bench presses was associated with a lower risk of incident MetS (HR: 0.66 [0.50–0.86]) while compared with the lowest muscular strength quartile during a mean follow‐up period of 6.7 years.^13^ Their findings were consistent with the present cohort study despite no adjustment for baseline PA levels. Because there were no cohort studies for young adults to clarify the inverse association of MEC with incidences of MetS and related features, our study can fill the gap in knowledge and affirm the addictive effect of greater MEC on CRF to prevent the development of MetS in young adults.

Mechanisms have been proposed previously to account for resistance training and increased muscular strength with a reduction of abdominal fat and insulin resistance.^14^, ^15^ In addition, both dynamic *and isometric resistance training were found to improve endothelial function among healthy individuals* and those with CVD or metabolic disorders,^35^ and chronic resistance training rather than its acute effect could reduce arterial stiffness,^36^, ^37^ accounting for the effect of BP reduction and the addictive effect with CRF to reduce the risk of hypertension. Furthermore, chronic resistance training to increase MEC was noticed to reduce systemic inflammatory markers with body mass reduction, which may reduce TG and increase HDL‐C levels.^19^, ^38^ Mechanisms for the effect of greater CRF related to aerobic training on improving the established MetS and related components have been reasoned by significant reductions in systemic inflammation, insulin resistance, abdominal fat and body weight, which are similar to that for MEC.^31^, ^39^ Notably, the effect of CRF on reducing abdominal fat and body weight, the major MetS feature, was greater than the effect of MEC,^40^ probably accounting for the null association between MEC and incidence of MetS with simultaneous adjustments for CRF in this study.

### Limitations and strengths

To the best of our knowledge, this cohort study was the first report confirming an inverse association of MEC with the incidence of MetS with adjustments for several potential confounders, including PA levels in young adults. On the contrary, some limitations were present in this study. First, unmeasured confounders may have affected the results, although several covariates were adjusted for. Thus, future studies using randomized clinical trials are needed to confirm the causal association in young adults. Second, the findings may not be generalizable to all young adults, as our study participants were exclusively obtained from the relatively physically fit young military personnel. Third, the assessment of leisure‐time PA levels was dependent on questionnaires rather than precise measurements using pedometers and accelerometers, which might have a recall bias.

## Conclusions

This study suggests that although the protective effects of MEC to reduce the incidence of MetS and most of its related features were mainly driven by CRF in young adults, there was an addictive effect of greater MEC on CRF to prevent the development of new‐onset MetS before midlife. For clinical applications, it may be necessary to achieve greater levels of CRF and MEC among relatively healthy young adults independent of the PA levels, and this combined effort should be encouraged for the primary prevention of the early development of new‐onset MetS and its related metabolic disorders. The long‐term prevention of MetS started early in life could go a long way in the prevention of CVD and its associated higher mortality.

## Funding

The study was supported by the Medical Affairs Bureau Ministry of National Defense (MND‐MAB‐D‐113200) and the Hualien Armed Forces General Hospital (HAFGH‐D‐113008) grants, which was the main institution involved in the study design, data collection, analyses and writing of this research.

## Conflict of interest statement

The authors have no conflicts of interest to declare.

## Data Availability

The datasets generated and/or analysed during this study are not publicly available due to materials obtained from the military in Taiwan, which were confidential, but are available from the corresponding author on reasonable request.
